# A review of the drivers and barriers influencing the impact of Water, Sanitation and Hygiene on attendance and retention of pupils in primary schools in developing countries

**DOI:** 10.3389/fpubh.2026.1804891

**Published:** 2026-08-12

**Authors:** Esther Adekunle, Ferdinand Ottoh, Goddy Osimen, Adedotun Akinola

**Affiliations:** 1 Department of Political Science and International Relations, Covenant University, Ota, Nigeria; 2 Department of Political Science, University of Lagos, Akoka, Yaba, Nigeria; 3 Department of Architecture, Covenant University, Ota, Nigeria

**Keywords:** attendance, barriers, drivers, retention, SDGs4, SDGs6, sustainable development, WASH

## Abstract

Drivers and barriers influencing the impact of Water, Sanitation and Hygiene (WASH) on attendance and retention of pupils in schools in developing countries were examined in this study. A Narrative review was adopted for this study which involved a Literature search of literature published between 2015 and 2025. The study identified Water Availability, Sanitation access, Hygiene Practices, Health improvements, Community involvement, Menstrual Hygiene Management (MHM) Support, Policy and funding support, Behavior change and Socio-economic context as the drivers of the impact of WASH on attendance and retention of pupils in primary schools in developing countries. Also, Inadequate Infrastructure, Poor design of infrastructure, Poor Maintenance of facilities, Overcrowding, Lack of Menstrual Hygiene Management (MHM) Facilities, Cultural and Social Norms, Low awareness and Poor Hygiene Behavior, Financial Constraints, Weak Institutional and Policy Support, Environmental Challenges, Household Responsibilities and socio-economic factors and Poor monitoring and evaluation were identified as barriers of the impact of WASH on attendance and retention of pupils in primary schools in developing countries. The study recommends that the collaboration of governments, civil societies, private sectors, and research institutions is crucial as far as dealing with political, economic, and other underlying factors of WASH policies in primary schools in developing countries. This study will provide empirical data for researchers and policy makers towards achieving Sustainable Development Goals 4.

## 1 Introduction

Access to quality education is a basic human right and a major driver of socio-economic growth (1). Unfortunately, in various developing states, the rate at which children attend and get enrolled in schools is lower than what is acceptable owing to various health, social, and environmental factors (2). Among the major drivers that have been identified in relation to accessing educational institutions is the availability of Water, Sanitation, and Hygiene (WASH) services at schools (3, 4). The provision of WASH, including safe drinking water, proper sanitation, hand-washing facilities, and hygiene education, has been identified as a major driver that sustains a healthy learning environment (5). In this regard, while WASH is creating a platform to shield students against communicable diseases, it is also enhancing their comfort and safety (6). Incentives like access to water, sanitation facilities, health promotion activities, Menstrual Hygiene Management (MHM) practices, community participation, and supportive policies enhance the connection between WASH and education because they increase the chances that a child is not going to be absent due to illness (7–9). However, some factors such as a lack of infrastructure and infrastructure maintenance influence WASH and could limit it from playing its effective role in enhancing school attendance and retention (10). Nevertheless, some conditions such as poverty and water scarcity mediate and affect the association between WASH being conveyed and playing a role in providing benefits in education (9).

In most developing countries, WASH situation is better in secondary schools (11) and much better in tertiary institutions (12) compared to primary schools/basic education institutions (13). Therefore, this study is focused on WASH in primary schools. Despite the awareness of the relevance of WASH, available evidence suggests that the impact of WASH in terms of attendance, retention and enrollment does not have some form of consistency and generality (14). While in some of the institutions the improvement in the level of student participation post-WASH programs is significant (10), other institutions are faced with high rates of absenteeism and dropout programs (15). Knowledge concerning factors that trigger increased effects of WASH and factors that impede such effects is fundamental in developing programs that address these effects in a context-dependent strategy in maximizing their gains in educational endeavors. It is imperative to have an equity-based perspective that will allow one to analyze how interventions of WASH within schools impact the presence and persistence of the learners within developing nations. WASH issues can affect learners differently based on various factors including disability and socioeconomic status. This paper discusses drivers and barriers of the impact of WASH on attendance and retention of pupils in schools in developing countries. Therefore, the objectives of this article are to:

(i) Analyze drivers of the impact of WASH on attendance and retention of pupils in schools in developing countries.
(ii) Analyze barriers of the impact of WASH on attendance and retention of pupils in schools in developing countries.

The present review is linked to Sustainable Development Goals (SDGs) 4 (Quality Education) and 6 (Clean Water and Sanitation) as it assesses the effect of interventions related to Water, Sanitation and Hygiene (WASH) on attendance and retention rates of pupils in primary schools in developing countries. Availability of WASH facilities facilitates healthy and better learning environments, especially in those settings where access is limited. The current review brings together various drivers and barriers for WASH interventions in schools based on evidence to identify the factors which either hinder or enable them. The results obtained are useful for policy making and include recommendations on enhancing collaboration between sectors involved (education, health, and WASH), as well as financing and governance. The study adds value to the existing literature by bringing together different kinds of evidence into a common framework of factors influencing WASH interventions.

## 2 Methods

In this study, a narrative literature review was employed to explore the factors driving and hindering the effect of Water, Sanitation and Hygiene (WASH) programs on attendance and retention of students in primary schools in developing countries. The use of a narrative literature review is justified by the fact that the research will seek to make sense, synthesize, and generate conceptualization of knowledge from the diverse pool of literature instead of synthesizing the evidence as would have been expected in a systematic review [(16–18, 63, 19)]. Narrative reviews were preferred to systematic reviews for this multidisciplinary topic because they offer flexibility in including literature from various sources to achieve a broader picture of the factors affecting the efficacy of WASH programs in schools. However, narrative reviews are more interpretive and hence vulnerable to both selection and interpretation biases (62, 64).

A thorough review of literature was carried out via PubMed, Google Scholar, and Scopus. The literature review involved a combination of keywords that relate to WASH and its implementation, which included WASH, policy, implementation, framework, driver, barrier, challenge, and scale-up, alongside keywords that relate to the educational setting, such as primary schools, school attendance, school retention, basic education, quality education, rural schools, informal settlements, urban poor, and developing countries. In order to identify information on policies and their implementation from sources that were not accessible via scholarly literature searches, grey literature was sought via institutional websites like the World Bank, WHO, UN-Habitat, WSUP, USAID, IRC, and others.

The literature search was restricted to publications written in English from 2015 to 2025 to ensure that the review contained up-to-date information on school WASH programs and policies in developing nations. The relevant sources were limited to peer-reviewed articles, review articles, policy papers, technical reports, and institutional papers that evaluated the effect of school WASH on pupils’ attendance and retention or identified issues related to the implementation and sustainability of school WASH programs. Literature was not considered if it only targeted high income countries, home WASH not involving school, learning environments other than primary schools, publications before and after the specified period, conference papers without full texts, or subjects unrelated to this review.

A total of about 180 articles were retrieved. After the elimination of duplicates found in the various databases, about 150 unique articles remained. Titles and abstracts of the articles were then reviewed to establish whether they were related to the objectives of the review before assessing their full texts. This was done by establishing whether the article focused on school-based WASH programs implemented in primary schools in developing countries and the impact that these programs had on attendance and retention or implementation. About 70 sources were selected for the synthesis.

Data analysis was conducted using inductive thematic approach. Rather than using themes in advance, the iterative review of the selected literature enabled recurring patterns and themes to emerge from the findings reported across the studies. Two key themes, interrelated with each other, were found to be prevailing within the data gathered from the literature. The first one is drivers, meaning the factors that positively contribute to the implementation of WASH interventions in schools and their positive impact on pupils’ attendance and retention. The second theme is barriers which refer to institutional, economic, infrastructural, behavioral, and policy-related factors that hinder the effective implementation of WASH interventions and their long-term impact on the pupils’ attendance and retention in primary schools in developing countries. Thus, thematic categories used in the synthesis have been developed on the basis of the data gathered from the literature rather than predetermined beforehand to ensure the coherence of the synthesis. Narrative synthesis approaches have been previously used in different studies (19–24).

## 3 Results

### 3.1 Drivers of the impact of WASH on attendance and retention of pupils in primary schools in developing countries

#### 3.1.1 Water availability

Provision of safe drinking water in schools is a universal factor contributing to school attendance and retention (10, 25). This is because with the availability of clean water, students will not contract waterborne illnesses like diarrhea, cholera, or typhoid fever, which rank among the main causes of students not attending school (13, 25). Access to water will also enable good personal and school hygiene habits like frequent hand washing or usage of toilets (26). In rural areas, the absence of water means that students, particularly girls, will not attend school because they will be needed at home to provide this vital commodity (27). Therefore, the provision of water in schools will help eliminate this problem.

#### 3.1.2 Sanitation access

Providing adequate and appropriate sanitation facilities is a key mandate that ensures comfort, privacy, and dignity levels are maintained for school-going children (28, 29). Providing girls and boys with separated sanitation facilities makes them stay in school for the whole day (7). In girls’ cases, a lack of appropriate toilet facilities results in absenteeism due to a lack of private toilet facilities that would enable them to stay in school (30). The schools should provide proper sanitaries so that a favorable environment may be created.

#### 3.1.3 Hygiene practices

Hygiene practices, including hand-washing with soap, play a key consideration in ensuring that the spread of communicable diseases is reduced in learning institutions (13, 31, 32). With hygienic practices among school-going children, there is a great decrease in cases of illnesses such as respiratory and intestinal ailments. This effect bears a direct relationship to an increase in the number of days not missed at school. Hygiene practices play an important behavioral aspect in emphasizing the positive effect of WASH among those in attendance.

#### 3.1.4 Health improvements

Health outcomes improvement is one of the most visible channels through which the education sector is affected by WASH (3). Providing children with safe drinking Water, Sanitation and Hygiene facilities translates to healthier children who can physically go to school more often. Healthier children display stronger concentration levels, higher levels of energy, and stronger engagement capabilities with the learning processes.

#### 3.1.5 Menstrual hygiene management (MHM) support

Providing menstrual hygiene management facilities plays a significant role in the successful education of girls (33). Many developing nations have witnessed a high dropout rate of girl adolescents from schools because of the lack of privacy, water, and disposal amenities in schools (34). Providing girl students with female-friendly toilets, water, and safe change rooms for changing sanitary towels will increase their confidence levels and make them feel free and safe in schools (35).

#### 3.1.6 Safe and supportive school environment

Hygienic conditions that are neat and safe influence pupils to regularly attend school. It further encourages parents to send their children to school (3). Schools that have effectively functioning WASH facilities have a positive perception of being caring institutions concerned about children’s health (26). Hygienic conditions make a pupil feel safe without fears of being embarrassed due to poor sanitation.

#### 3.1.7 Community involvement

Community involvement in school WASH interventions may be considered the most important factor affecting the sustainability and effectiveness of such projects. Involving parents and the community at large in the implementation of WASH projects, such as the construction and maintenance of WASH facilities, increases responsibility and ownership of such projects (9). Furthermore, the participation of the community is an excellent way of reinforcing good habits acquired at school.

#### 3.1.8 Policy and funding support

The sustainability of WASH interventions in schools requires a well-strategized policy and financing component. Commitment by the government provides for development of standards, financing, and maintenance of facilities (9, 36). It was observed that the unavailability of financing and infrastructure creates conditions for WASH infrastructure to fall into disuse. This, in turn, ensures sustainability of the WASH services provided, translated through improvements in school attendance and enrollment.

#### 3.1.9 Behavior change

Programs that aim to transform behaviors, like those that promote cleanliness, make physical infrastructural developments associated with WASH more efficient. Since children would understand the importance of hand washing, toilets, as well as how to handle water, they will conduct themselves in a way that ensures they are always guarded against diseases (37, 38). Behavior change initiatives are key to developing lasting benefits not only in the walls of the learning facility but also in the home setting, which ensures that more students attend school.

#### 3.1.10 Socio-economic context

The overall social and economic context affects the extent to which the impact of WASH on education participation is realized. Poverty levels and education attainment among parents in society are some factors that determine to what extent children are able to take full advantage of WASH services offered to them (39). Societies with high costs associated with living may see their overall costs related to diseases reduced by improved WASH facilities.

Taken together, these factors make it clear that WASH can increase school attendance and enrollment through various channels, such as health, dignity, enabling environments, and enabling institutions (40). When these factors act together, the tools that WASH program interventions provide are extremely useful in developing countries to make educational resources more accessible. Table 1 shows summary of key drivers of the impact of WASH on attendance and retention of pupils in schools in developing countries.

**Table 1:** Summary of key drivers of the impact of WASH on attendance and enrolments of pupils in primary schools in developing countries.

S/N	Driver	How it affects attendance and enrolment
1	Water availability	Reduces illness, improve attendance
2	Sanitation access	Prevents gender-based absences
3	Hygiene practices	Reduces disease spread
4	Health improvements	More consistent attendance
5	MHM support	Keep girls in school
6	Safe environment	Encourages enrolment
7	Community involvement	Sustains improvements
8	Policy and funding	Ensures lasting impact
9	Behavior change	Creates lasing benefits
10	Socio-economic context	Modulates overall effects

### 3.2 Barriers of the impact of WASH on attendance and retention of pupils in primary schools in developing countries

#### 3.2.1 Inadequate infrastructure

One of the biggest hindrances to the impact of WASH on education is the weakness of the infrastructure and the condition of the infrastructure provided in the schools (10). Infrastructure such as toilets and water points is either lacking or inadequate in schools (41). Also, the number of toilets or water points is very low and causes queues and dissatisfaction to the point where absenteeism and low retention are seen, particularly in the case of girls (7).

#### 3.2.2 Poor design of infrastructure

Even where infrastructure is available, the design of the infrastructure is poor (42). Poor infrastructural design have impact on accessibility, acceptability and usage. Pupils might opt to stay away from school because of the unhygienic conditions as a result of poor design infrastructure (9).

#### 3.2.3 Poor maintenance of facilities

Even when these WASH facilities are offered in the first place, poor maintenance practices result in these facilities not functioning in the long run (9). Non-functional taps, blocked toilets, a missing soap component, as well as a missing water component in either washing hands or flushing toilets, are enough to deter students in institutions from going to these facilities (9, 43). This not only affects hygiene standards but also affects students’ confidence in their environment.

#### 3.2.4 Overcrowding

High ratios of students to toilets contribute to overcrowding (9, 13). Also, inadequate sanitation facilities contribute to the problem. When students are denied adequate resources, the results are increased waiting times and the risk of poor sanitation (9). Overcrowding can be a major factor affecting the attendance of adolescent girls (29). Overcrowding can lead to unhygienic conditions and thus contributing to attendance and retention of pupils. In the same breadth, overcrowding at hand wash stations also contribute to unhygienic/unsightly scenes leading to none usage of the facilities.

#### 3.2.5 Lack of menstrual hygiene management (MHM) facilities

The lack of MHM support creates a huge constraint for female students. Schools lacking private toilets, access to running water, bins for disposal, and materials for managing menses hinder young girls’ capability to cope with their monthly cycles (44, 45). This results in girls missing school for a few days in a month and, in the long term, dropping out completely. Improvements in WASH alone are not enough for equal school participation among girls and boys in the absence of MHM support.

#### 3.2.6 Cultural and social norms

Socio-cultural factors also impede the efficiency of WASH interventions. There are cases in which the issue of menstrual and sanitation taboos prevents the use of school toilets and hygiene facilities (46). Gender roles can prevent boys and girls from having equal educational opportunities by giving importance to boys’ schooling (47). Moreover, any negative attitudes regarding hygiene practices can affect the use of hygiene facilities by the students.

#### 3.2.7 Low awareness and poor hygiene behavior

In spite of the existence of WASH facilities in schools, poor hygiene practices and awareness among learners and educators may hamper the success of such facilities (10). Children may not be in the habit of washing their hands or using toilets in a proper manner to maximize the effects of such practices on the prevention of contagious diseases, thus promoting school attendance (48).

#### 3.2.8 Financial constraints

Lack of funding is a constraint that has hampered WASH programs. Many institutions cannot financially cater to the construction or replacement of WASH facilities (9). Additionally, government funding may not be adequate or reliable. Lack of funding is also a constraint that affects access to sanitation facilities. With programs that lack funding, even programs that have a positive influence on attendance may not continue to have a significant impact on retention.

#### 3.2.9 Weak institutional and policy support

The lack of adequate frameworks can affect the sustainability of WASH initiatives. With the failure to coordinate WASH and education, the failure to establish monitoring standards, and the failure to establish the party responsible for maintaining facilities, the facilities are not maintained as they should be. However, it is important to note that WASH facilities in themselves are not sufficient. In the absence of proper policies, funding, management, and accountability, WASH facilities will not operate at optimal capacity and when they fail to operate, students will not come to school or will leave school (36).

#### 3.2.10 Environmental challenges

Droughts, water shortage seasons, as well as difficult geographical environments, are some environmental constraints (49, 50). The health of pupils is at risk as a result of a lack of proper hygiene habits, something that is not possible within a school with unreliable water supplies, which would not function on its own WASH facilities (51, 52). The capacity of the intervention to improve the aspect of attendance is, thus, diminished.

#### 3.2.11 Household responsibilities and socio-economic factors

There are also broader socio-economic factors that can impact the efficacy of WASH at the school level (14). Children and girls, in particular, may have reasons to absent themselves from the educational institutions due to the lack of WASH at home. Also, the effects of WASH at the school level may be largely mitigated due to the limited availability of time due to poverty (3).

#### 3.2.12 Poor monitoring and evaluation

Lastly, the absence of monitoring and evaluation makes it difficult to know whether school WASH facilities are functional. Without data, there are no repairs or replacements when toilets are not functioning or are underutilized. Therefore, there are no modifications made to optimize educational goals (53).

Available literature reveals that factors inhibiting the achievement of WASH are complex, ranging from the infrastructure and maintenance gaps, socio-cultural factors, costs, and environmental constraints. This hampers the effectiveness of WASH in enhancing school attendance and enrollment, and hence the need for the various factors to be considered and addressed together with the provision of WASH infrastructure. Table 2 shows summary of key barriers of the impact of WASH on attendance and retention of pupils in schools in developing countries.

**Table 2:** Summary of key barriers of the impact of WASH on attendance and enrolments of pupils in primary schools in developing countries.

S/N	Barrier type	Main challenge
1	Infrastructure	Insufficient or non-functional facilities or poor design of facilities
2	Maintenance	Lack of repairs and supplies
3	Cultural factors	Social norms limiting use
4	Financial	Inadequate funding
5	Gender Issues	Lack of MHM support
6	Environmental	Water scarcity
7	Institutional	Weak policies and coordiantion
8	Behavioral	Poor hygiene practices
9	Monitoring	Lack of data and accountability

## 4 Discussion

WASH programs form a key part of the determination of participation in educational programs in developing countries. There is support that WASH practices have a symbiotic relationship with education programs. These are influenced by driving forces and inhibiting barriers. These driving forces increase interactions related to access to WASH programs and education. They do this by improving health, dignity, safety, and standards of the learning surroundings. There is evidence that access to safe drinking water in learning centers leads to a reduction in cases of water-related diseases such as diarrhea, cholera, and intestinal (9, 13). These are some of the primary causes of absenteeism in learning institutions. Healthy children are expected to attend school on a regular basis. They are expected to focus on everything that is happening in class. They are expected to be in school for a much longer time (54, 55).

Moreover, the role of functional hygienic facilities, illustrated by hand-washing facilities with soap, is emphasized by the reality that school attendance can be substantially enhanced by such facilities. Hand washing is effective in reducing the spread of contagious diseases, and hence reducing absenteeism due to illnesses is also promoted (56). Education initiatives on hygiene behaviors are also important for ensuring that hygiene facilities are used. Gender-sensitive WASH service delivery, such as menstrual hygiene management facilities, has been cited as important factors for girls’ education (9, 33, 34). It has been observed that toilet facilities that are attended to, water for washhands, or facilities for disposal of matter to be disposed of have been found to assist girls to stay in school (57).

However, this is not the case, there are some authors (9, 13) who have outlined some of the challenges which prevent WASH from playing its part in improving education outcomes. Among these is a lack of infrastructure support in institutions to offer WASH services (9, 13). Many of these institutions are not equipped with sufficient toilets or facilities such as water and hand-washing facilities. Even if these facilities are available in institutions, it becomes difficult to access them after a period of time when, for instance, the water tap might not be functioning or when toilets are blocked and without soap and towels (58).

Also, cultural as well as social issues act as an impediment. In many societies, the cultural perception concerning hygiene and menstrual issues influences the usability of the enhanced facilities offered by WASH programs, particularly among the female population at schools (46). Also, home chores, which include collecting water or caring for a sick member, as a rule, keeps children from attending classes, thus making the effectiveness of the enhanced facilities by WASH programs ineffectual. A lack of funding as well as ineffectuality is a significant impediment to the effectiveness of the work being carried out by the programs offered by the WASH initiative (9, 59).

Environmental constraints such as a lack of water supply or seasonal droughts constrain the use of WASH facilities (49, 50). In arid or semi-arid regions, for instance, a lack of access to a constant supply of water means that even flush toilets or handwashing facilities built to high standards cannot function properly (60, 61). In addition, weak monitoring and evaluation systems limit the tracking of facility conditions or the measurement of its influence on attendance and retention (52).

Based on the foregoing, it can be said from the available literature that while WASH has huge potential in improving attendance and enrollment in schools, it should also focus on overcoming barriers. To ensure WASH makes a huge contribution towards participation in education, it is necessary to leverage the powerful drivers in WASH to ensure behavioral changes in addition to infrastructure development. Apart from this, it is also important to ensure changes in institutions and infrastructure to ensure WASH programs reach their full potential for improving access to education in developing countries. Table 3 shows summary of Drivers (Positive factors) and barriers of WASH impact on School Attendance and retention.

**Table 3:** Summary of drivers (positive factors) and barriers of WASH impact on school attendance and enrolments of pupils in primary schools in developing countries.

S/N	Dimension	Key drivers (positive factors)	Key barriers (limiting factors)
1	Water supply	Having clean and accessible drinking water can help prevent sickness and absenteeisms among children	Water scarcity, seasonal water shortage, and inadequate water supply affect the usability of the facilities.
2	Sanitation facilities	The provision of clean, adequate, and private toilet facilities promotes frequent attendance	Inadequate, unclean, or non-functional toilets will reduce school attendance
3	Hygiene facilities	Hand-washing stations with soap promote cleanliness and prevent infections	When soap, water, or hand washing is not available, the limiting factor for the behavior change process
4	Health outcomes	Less diarrhea and diseases translate to improved physical health among pupils to attend school	Poor WASH increases the burden of diseases, which leads to absenteeism
5	Gender considerations	Providing gender-specific toilet facilities as well as MHM support leads to increased enrolment of girls in schools	No privacy and menstrual facilities are associated with girl absenteeism and dropouts
6	School environment	Hygienic and safe learning surroundings encourage parents to send their children	Poorly maintained and unsanitary facilities encourage fear, discomfort, and stigma
7	Hygiene education	Behavior change communication supports long-term use of WASH services	Lack of awareness and bad hygiene habits inhibit the use of available facilities
8	Community engagement	Community participation is critical in ensuring the ownership and sustainability of WASH services	Lack of active involvement within a community can result in a lack of attention and maintenance of facilities
9	Policy and funding	Investment by the government and the establishment of standards ensure the long-run provision of WASH basics	Insufficient funding & weak institutional arrangements are hindrances for implementation
10	Monitoring systems	Good monitoring is the way to ensure maintenance as well as accountability	Absence of data and monitoring makes it impossible to increase services
11	Socio-cultural factors	Promoting positive attitudes towards sanitation and the education of girls will have a positive impact on attendance	Cultural taboos and gender biases limit access to WASH facilities
12	Household context	Enhanced home hygiene interventions support school WASH programs	Children miss school because of water collection responsibilities and domestic sanitation challenges

### 4.1 Limitation of this study

There are certain limitations associated with this study that need to be taken into consideration when making sense of the results. First, the use of the narrative review method, even though appropriate for integrating a variety of information related to the multi-faceted nature of the discussed topic, implies an element of interpretation by the researcher and is likely to be susceptible to selection and publication bias compared to systematic review methodologies. The second limitation is associated with the focus of the review on the English-language literature from 2015 to 2025, and hence, certain pieces of evidence might have been overlooked. The third limitation is associated with the fact that the literature reviewed reflected the realities of different countries in terms of socio-economic and political factors as well as different WASH programs and their implementation. Finally, differences in study designs, outcome measures and definitions of attendance, retention and WASH interventions used in each source might have affected the synthesis of evidence.

## 5 Conclusion

Although the world has made progress in Water, Sanitation and Hygiene (WASH), unequal distribution of quality WASH in schools in low and middle-income countries, especially in the informal settlements of urban areas, remains a concern for attendance and enrollment rates in schools. It requires a multi-sectoral approach in which the forces driving equitable WASH policy can be strengthened while the hindrances can be addressed from the bottom up through community engagement and capacity development at the local level via policies from the top down.

The role of researchers cannot be overstated as far as providing context-specific information and visual representation of evidence about WASH and educational outcomes is concerned. The collaboration of governments, civil societies, private sectors, and research institutions is crucial as far as dealing with political, economic, and other underlying factors of WASH policies is required. The partnership of research institutions can prove instrumental for developing interventions related to WASH for improving attendance rates and retention as well as learning outcomes for disadvantaged students.
